# Introduction of a bone-centered three-dimensional coordinate system enables computed tomographic canine femoral angle measurements independent of positioning

**DOI:** 10.3389/fvets.2022.1019215

**Published:** 2022-11-24

**Authors:** Andreas Brühschwein, Bronson Schmitz, Martin Zöllner, Sven Reese, Andrea Meyer-Lindenberg

**Affiliations:** ^1^Clinic of Small Animal Surgery and Reproduction, Veterinary Faculty, Centre of Veterinary Clinical Medicine, Ludwig-Maximilians-Universität München Munich, Munich, Germany; ^2^Department of Veterinary Sciences, Veterinary Faculty, Institute of Veterinary Anatomy, Histology and Embryology, Ludwig-Maximilians-Universität München Munich, Munich, Germany

**Keywords:** dog, femur, computed tomography, 3D coordinate system, angular deformity, torsion, varus

## Abstract

**Introduction:**

Measurement of torsional deformities and varus alignment in the canine femur is clinically and surgically important but difficult. Computed tomography (CT) generates true three-dimensional (3D) information and is used to overcome the limitations of radiography. The 3D CT images can be rotated freely, but the final view for angle measurements remains a subjective variable decision, especially in severe and complex angular and torsional deformities. The aim of this study was the development of a technique to measure femoral angles in a truly three-dimensional way, independent of femoral positioning.

**Methods:**

To be able to set reference points in any image and at arbitrary positions of the CT series, the 3D coordinates of the reference points were used for mathematical calculation of the angle measurements using the 3D medical imaging Software VoXim^®^. Anatomical reference points were described in multiplanar reconstructions and volume rendering CT. A 3D bone-centered coordinate system was introduced and aligned with the anatomical planes of the femur. For torsion angle measurements, the transverse projection plane was mathematically defined by orthogonality to the longitudinal diaphyseal axis. For varus angle measurements, the dorsal plane was defined by a femoral retrocondylar axis. Independence positioning was tested by comparison of angle measurement results in repeated scans of 13 femur bones in different parallel and two double oblique (15/45°) positions in the gantry. Femoralvarus (or valgus), neck version (torsion), and inclination angles were measured, each in two variations.

**Results:**

Resulting mean differences ranged between –0.9° and 1.3° for all six determined types of angles and in a difference of <1° for 17 out of 18 comparisons by subtraction of the mean angles between different positions, with one outlier of 1.3°. Intra- and inter-observer agreements determined by repeated measurements resulted in coefficients of variation for repeated measurements between 0.2 and 13.5%.

**Discussion:**

The introduction of a bone-centered 3D coordinate system and mathematical definition of projection planes enabled 3D CT measurements of canine femoral varus and neck version and inclination angles. Agreement between angular measurements results of bones scanned in different positions on the CT table demonstrated that the technique is independent of femoral positioning.

## Introduction

### Clinical relevance

In the canine femur, determination of alignment and torsional deformities is clinically and surgically important but difficult. Diagnostic evaluation of morphology and deformation of the canine femur is commonly determined by angular measurements in radiographs (1–7).

### Principles and geometry of radiography

Plain projectional radiography inheres in the transformation and reduction of a three-dimensional (3D) object into a two-dimensional (2D) image (8). Summation of spatial information along the path of the x-ray beam projects all structures on top of each other into one single plane (8). Superimposition, magnification, and distortion cause geometric error (8). Distortion is unequal magnification and creates a radiographic image that does not truly represent the real shape and size of the examined object (8). Angular measurements require two lines intersecting in the same plane, which is called coplanarity (9). Axes in the 3D patient are skew lines that become coplanar due to projection into a 2D image. Skew lines lie in different planes, share no intersection, and do not form an angle in three-dimensional geometry (10).

### Limitations of radiography

Measurement error can be created by magnification, distortion, and variable projection of skew lines into the image plane (8). In radiography, identically constant patient positioning, X-ray beam centering, and alignment are aimed to standardize geometric distortion and minimize inherent radiographic limitations (8). Radiographic angle measurement techniques are highly dependent on standardized positioning, and minor deviations in the positioning of the limbs can lead to variation in the results of angle measurements (11–17). To improve radiographic positioning, especially for difficult projections, fluoroscopic guidance can be used if available (18). Many radiographic and computed tomographic techniques were developed in normal cadavers or single bones (15–32). However, femoral angle measurements are especially interesting in canine patients with patellar luxation (1, 3, 5–7, 19, 33–45) or severe post-traumatic bone deformity (4, 13, 46, 47). The positioning of isolated normal bones in a research setting differs from the positioning of canine patients with bone deformities and limited articular range of motion due to osteoarthritis or muscular contractions in clinical practice. During radiography of the canine femur in a craniocaudal view, slight variations in limb positioning or beam alignment may influence the measurement results of the distal femoral varus angle in an unpredictable manner (15–17).

### Canine femoral deformities

In a *valgus* deformity, the distal part of the limb deviates away from the midline with lateral angulation along the dorsal (frontal) plane, and the medial deviation is termed *varus* (15, 17). The *dorsal plane* is parallel to the dorsal surface of the body or body part and perpendicular to the *sagittal* (median and paramedian) and *transverse planes* (48, 49). The use of the synonymous term frontal plane is not recommended by the current Nomina Anatomica Veterinaria (48). The canine *femoral neck version* refers to the orientation of the femoral neck in relation to the condyles in a distoproximal view (18). *Femoral (neck) inclination* is the cervicodiaphyseal angle between the femoral neck and diaphyseal axis (23, 28, 50–52). To overcome the limitations of radiographic projections, biplanar methods with mathematical correction or calibration curves were used (20, 27, 53–55). In humans, *femoral torsion* due to diaphyseal twisting and femoral neck version can be discriminated (51). In dogs, no criteria are currently known that allow a distinction to be made, which is why these terms are often used synonymously (20, 22, 56). The normal femoral neck is oriented in craniomedial direction termed anteversion (18) and for caudal deviation, the term retroversion is used (56, 57). Normal canine anteversion (antetorsion) angles were reported to be between 12 and 40° (18). In a systematic review based on 29 individual studies, numerous standard values for canine anteversion angles and other femoral and tibial measurements in different breeds and with different imaging measurement methods were compiled (58).

### Computed tomography

Computed tomography (CT) generates true three-dimensional information and is used to overcome the limitations of two-dimensional radiographic projection errors (13, 14, 17, 19, 21, 22, 24, 25, 30, 40–43, 45, 47, 53, 55, 57, 59–65). As with radiography, precise and standardized positioning is also required for various CT-based measurement methods (21, 39, 40, 59). In complex hind limb deformities, a combined evaluation of the femur and tibia is commonly aimed. Canine stifle joints normally cannot be extended to 180 degrees, and at least one bone, femur, or tibia must be scanned obliquely, if not positioned and scanned individually. In clinical patients, perfectly extended, straight, and parallel limb positioning is often limited. In patients with severe fracture malunion or in patients with concurrent conditions that restrict the range of motion and interfere with straight positioning, standardized positioning is difficult and a technique that is completely independent of positioning would be helpful.

Usually, the reference points for angular measurements are located in different CT images, so postprocessing of cross-sectional images is required. CT measurements are performed in a single CT image or images generated by image fusion or overlay in a single superimposed image (24, 63, 66, 67), using multiplanar reconstruction (MPR) (21, 22, 40, 53), maximum intensity projection (MIP) (21), or volume rendering technique (VR) (13, 14, 17, 21, 22, 25, 43, 46, 47, 55–57, 59–63). MPR, MIP, or VR allow free choice of plane, rotation, and perspective, but after the final post-processing, the result is a flat 2D image that lacks the third dimension. The axes defining the angle are coplanar in the post-processed and reconstructed CT image, but remain oblique skew lines in the patient. The positioning of a normal bone can be standardized but becomes more difficult with increasing deformation (13). VR CT allows free 3D rotation of bones, which is similar to free virtual positioning. The selection of one single view on a bone, to create a final 2D projection for angular measurements, remains a subjective decision. This perspective can be standardized for normal bones, but angle measurements in severe and complex angular and torsional deformities of the canine femur are problematic despite the use of 3D VR CT (13).

### Study objectives

The goal of this study was the development of a CT-based technique to measure canine femoral varus (and valgus), neck inclination, and version (torsion) angles independent of femoral positioning in the CT scanner gantry in a truly three-dimensional way with the precise mathematical definition of projection planes and projected angles.

## Materials and methods

### Development of the technique

#### CT data

A CT bone scan of a presumably normal canine femur was queried and retrieved from the picture archiving and communication system (dicomPACS, Oehm & Rehbein, Rostock, Germany) of the small animal hospital. The hind limbs of this medium-sized mixed-breed dog were scanned for clinical reasons unrelated to the femur in a position that was similar to a ventrodorsal pelvic radiograph for canine hip dysplasia screening with extended coxofemoral, stifle, and tarsal joints. The dog had no history of hind limb lameness and an unremarkable clinical orthopedic examination based on archival clinical data in the hospital information system. The CT scan was performed with a helical multi-slice CT scanner with a fixed detector array design (Somatom Definition AS VA48A_02_P12, 64 Excel Ed. software Somaris/7 syngo CT VA48A Siemens Healthcare GmbH, Erlangen, Germany) in a helical mode. Scanning slice thickness was 0.6 mm, tube voltage 120 kV, tube rotation time 500 ms, spiral pitch factor 0.8, and X-ray tube current was 350 mA. A bone algorithm (deconvolution filter: kernel 70) was used to reconstruct slice thickness and increment to 0.6 mm, resulting in gap-free stacks of CT images.

#### Software

To truly measure selected clinically relevant morphometric angles in the canine femur in three dimensions, the anatomical axes, which are oblique skew lines, must be precisely defined in 3D space. Therefore, software with a 3D Cartesian coordinate system was a prerequisite and mathematical vector calculations were required to project 3D coordinates of anatomical reference points and axes into geometrically predefined planes. VoXim^®^ (version 6.5.1.1 (T2160910) Copyright, IVS Technology GmbH [LLC], Chemnitz, Germany) was used for the calculations and measurements. The software was designed for 3D image-guided surgery, had medical device approval, and was validated and used in prior studies (68–71). MPR, VR, bone segmentation, 3D coordinate system, and adjustable templates for angular calculations were the main features used in this project.

#### Implementation of three-dimensionality

Femoral reference points and axes described by radiography and computed tomography in a two-dimensional way were extended into a three-dimensional anatomical description using VR and orthogonal three-plane MPR. A bone-centered 3D coordinate system was introduced based on anatomical osseous reference points to enable angular measurements that are independent of femoral positioning on the CT table in the scanner gantry. To geometrically define transverse and dorsal projection planes for torsion and varus (or valgus) measurements in the 3D space, a mathematical definition based on anatomical reference points was used. 3D coordinates of the reference points were translated into the geometrically predefined projection planes. The mathematical definitions of the projection planes were inspired by anatomical cross-sectional planes, radiographic images, and X-ray beam projection techniques, as well as computed tomographic VR views.

### Description of anatomical reference points, axes, coordinate system, projection plane, and angular measurements

#### Proximal and distal reference points

**Figure 1:**
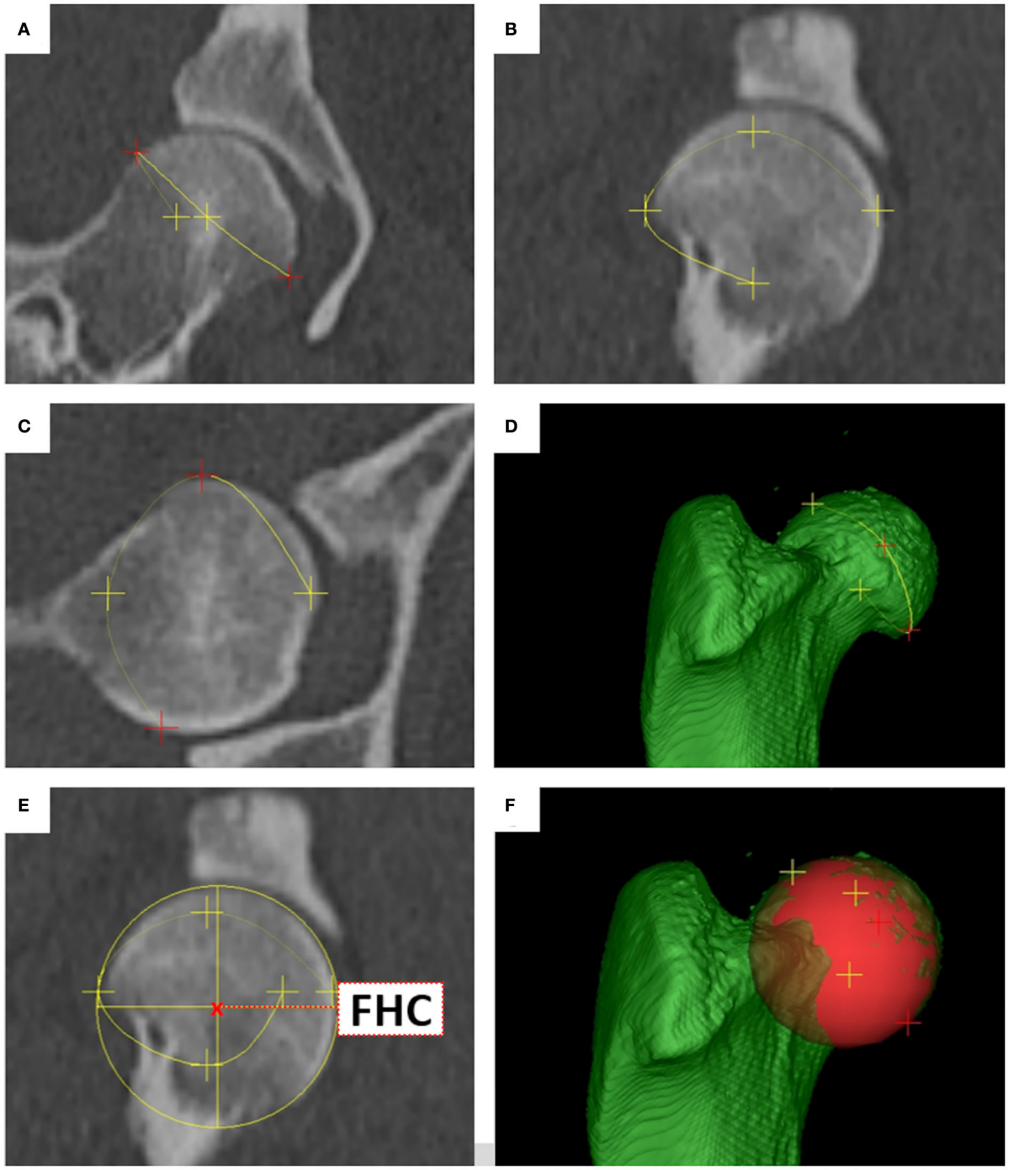
Femoral head center (FHC). Reference points **(A–C)** were placed along the bearing surface of the femoral head, forming a polyline **(A–F)** that encompassed the femoral head. The polyline triggered a fitting sphere **(E,F)** around the femoral head. The femoral head center (FHC) was the mathematical midpoint **(E)** of the 3D sphere, represented as a circle in MPR **(E)**, and ball in 3D VR **(F)**.

**Figure 2:**
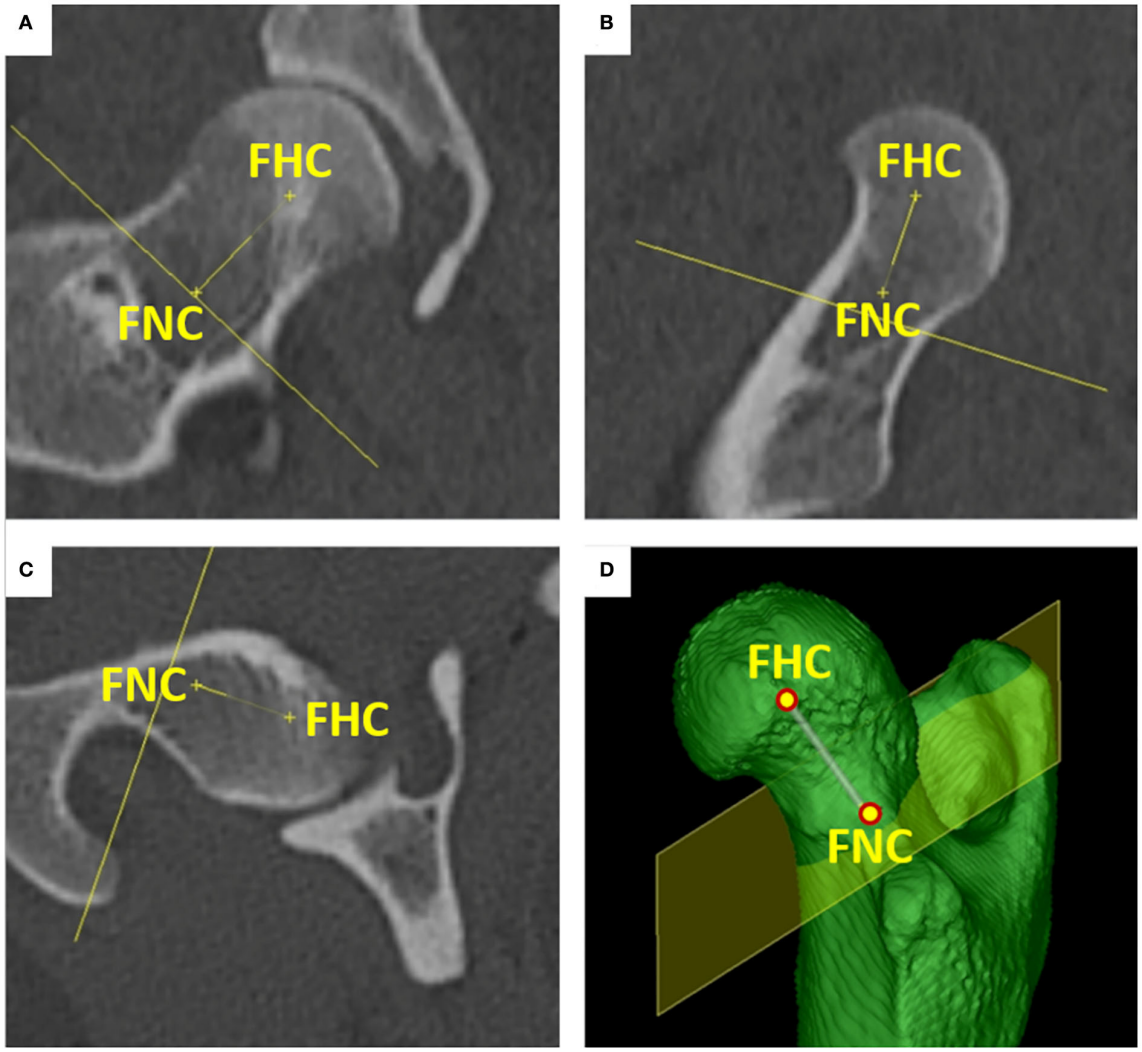
Femoral neck center (FNC). The femoral neck center was placed at the midpoint of the femoral neck isthmus. The point was set indirectly by moving a “virtual femoral neck resection plane” in a true transverse orientation to the femoral neck. A line was attached to the femoral head center (FHC) and orthogonally connected to the movable plane, with the femoral neck center (FNC) as the intersection point. The position of the femoral neck center was inspected and adjusted in orthogonal dorsal **(A)**, sagittal **(B)**, and transverse **(C)** planes and using multiple differently angled 3D VR projections **(D)**.

**Figure 3:**
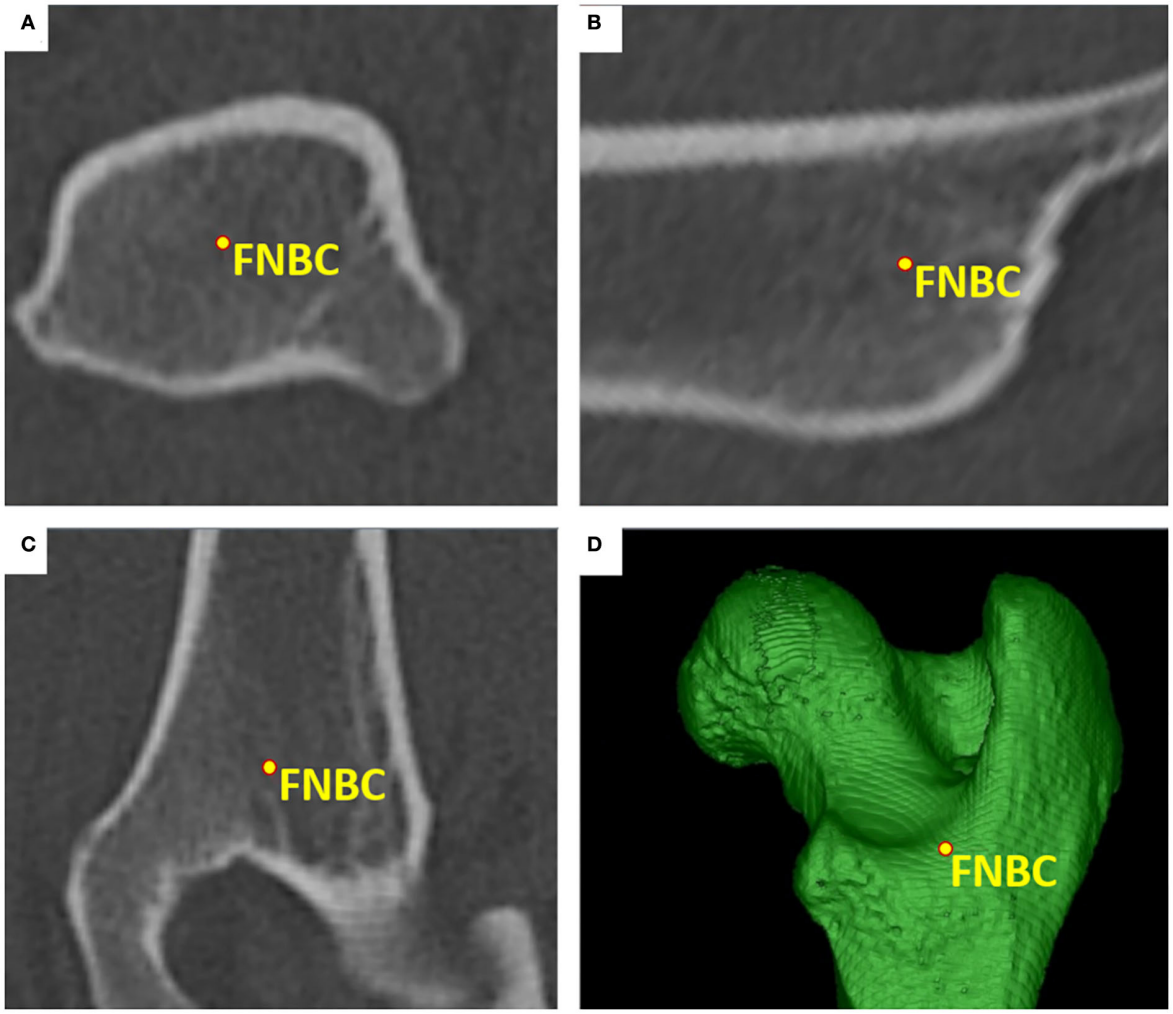
Femoral neck base center (FNBC). The femoral neck base center was the midpoint of the proximal femoral metaphysis at the level of the lesser trochanter. The position was determined visually using transverse **(A)**, sagittal **(B)**, and dorsal **(C)** planes, and multiple differently angled 3D VR projections **(D)**.

The *femoral head center* (FHC) was calculated as the midpoint of a 3D ball. Manually, the operator set various points at the subchondral bone surface of the femoral head along its load-bearing area excluding the capital fovea. The software automatically connected the individual points to a polyline and rendered a fitting sphere (Figure 1). The *femoral neck center* (FNC) was a semi-automatically determined point of intersection between a line originating from the femoral head center as perpendicular to a virtual movable plane, set transverse to the femoral neck at the level of its waist, resembling a virtual femoral neck resection. The desired midpoint at the isthmus of the femoral neck was verified in all three orthogonal MPR planes and multiple differently angled 3D VR projections (Figure 2). The *femoral neck base center* (FNBC) was set at the midpoint of the proximal femoral metaphysis in the transverse plane. The operator used an encircled crossline tool targeting the visually estimated midpoint of a cross-section image at the level of the highest elevation of the lesser trochanter. Verification of the position was performed using orthogonal MPR and multiple differently angled 3D VR projections (Figure 3).

**Figure 4:**
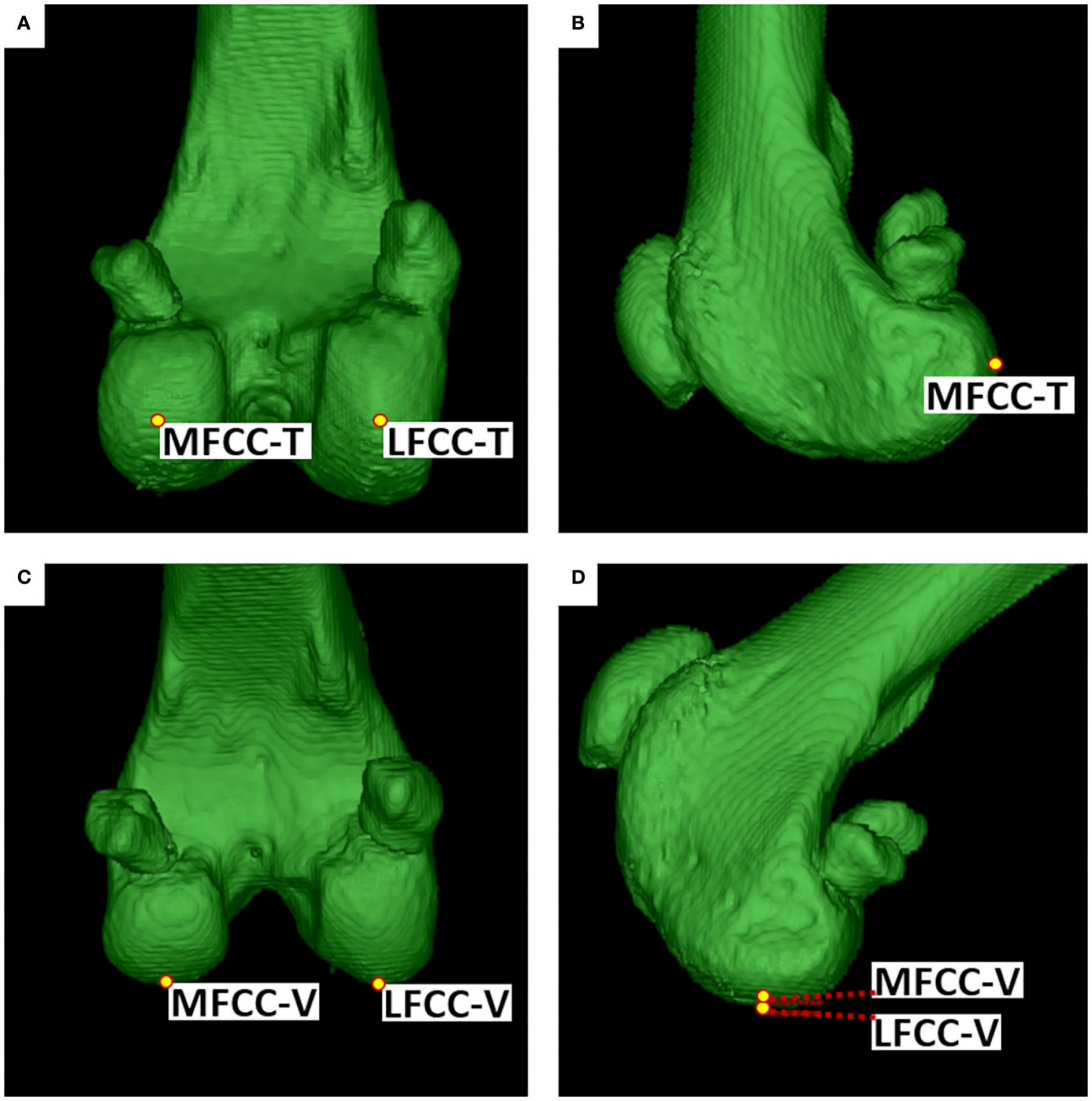
Femoral condyle centers (FCC). The medial (MFCC) and lateral femoral condyle centers (LFCC) were located at the caudal condylar aspect (MFCC-T & LFCC-T) for the determination of the torsion (T) angles **(A,B)** and distal condylar aspect (MFCC-V & LFCC-V) for varus or valgus (V) angles **(C,D)**. Points were set, corrected, and verified using MPR (not shown) and multiple differently angled 3D VR projections.

The *lateral femoral condyle center* (LFCC) and the *medial femoral condyle center* (MFCC) were the midpoints of the subchondral bone surface of the lateral and medial femoral condyle. Femoral condyle centers were set in the center of the convex joint surfaces at the most caudal aspects of the lateral (LFCC-T) and medial condyle (MFCC-T) to determine the femoral torsion (T) angles (syn. femoral neck version), and at the distal aspects of the lateral (LFCC-V) and medial femoral condyle centers (MFCC-V) for the varus and valgus (V) angles. To precisely place and align the retrocondylar tangents, medially and laterally at the same level of the femoral curvature, transverse, sagittal, and 3D views were used (Figure 4).

#### Proximal and distal axes

**Figure 5:**
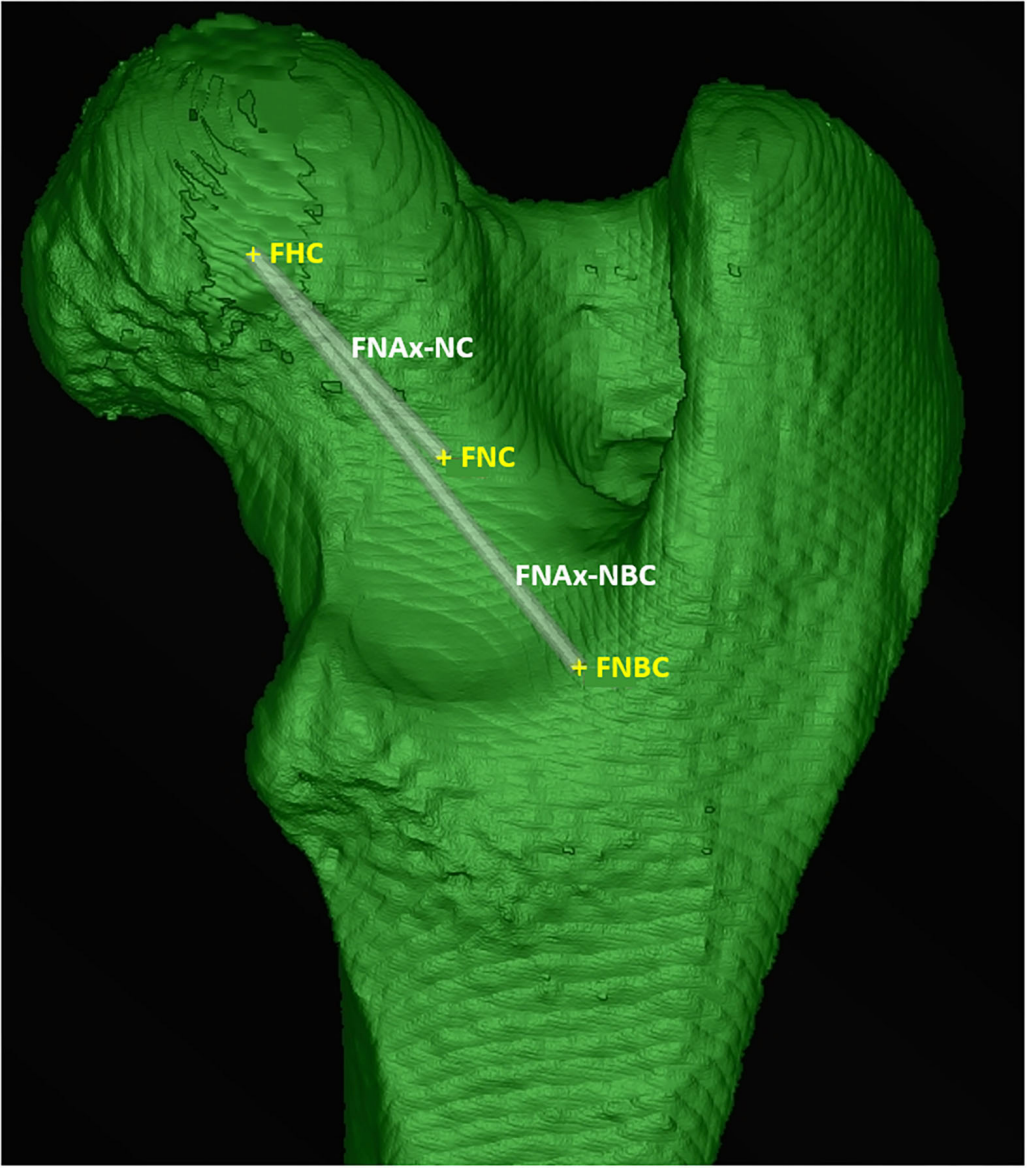
Femoral neck axes (FNAx). The femoral neck axis-neck center (FNAx-NC) connected the femoral head center (FHC) and the femoral neck center (FNC). The femoral neck axis-neck base center (FNAx-NBC) connected the femoral head center (FHC) and the femoral neck base center (FNBC).

**Figure 6:**
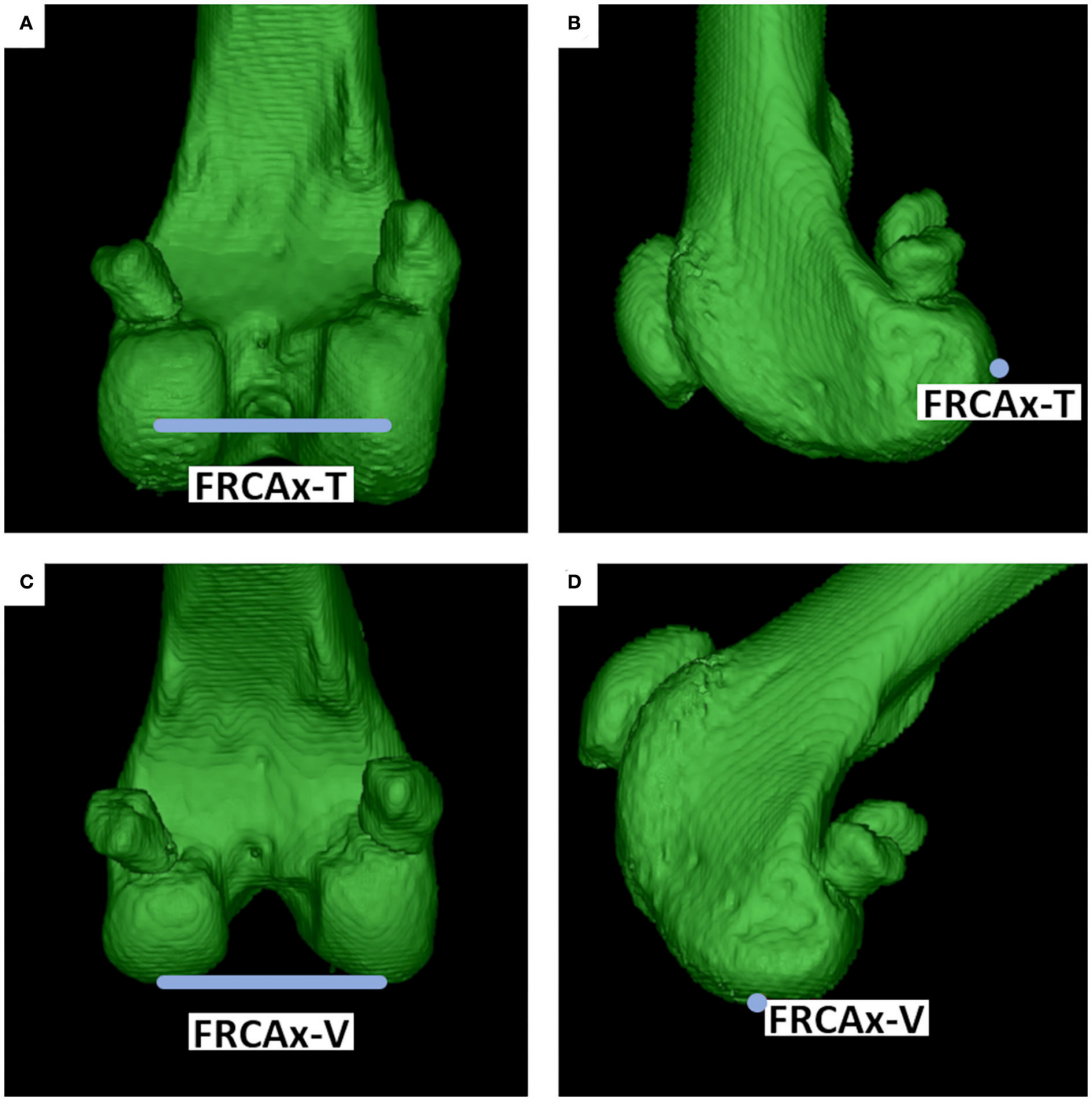
Femoral retrocondylar axes (FRCAx). The medial and lateral femoral condyle centers defined the femoral retrocondylar axes. FRCAx-T **(A,B)** for torsion angles was located at the caudal, and FRCAx-V **(C,D)** for varus or valgus angles at the distal condylar aspect of the femur.

A line connecting the femoral head center and the femoral neck center defined the *femoral neck axis-neck center* (FNAx-NC) for the calculation of the *femoral torsion angle-neck center* (FTA-NC) and the *femoral (neck) inclination angle-neck center* (FIA-NC). A line connecting the femoral head center and the femoral neck base center defined the alternative *femoral neck axis-neck base center* (FNAx-NBC) for the calculation of the alternative *femoral torsion angle-neck base center* (FTA-NBC) and the alternative *femoral (neck) inclination angle-neck base center* (FIA-NBC) (Figure 5). Two versions of a *femoral retrocondylar axis* (FRCAx) connected both centers of the subchondral bone surface of the lateral and medial femoral condyle creating a retrocondylar tangent, caudally (FRCAx-T) for the calculation of the femoral torsion angle and distally (FRCAx-V) for the varus (or valgus) angle (Figure 6).

#### Diaphyseal reference points and axes

**Figure 7:**
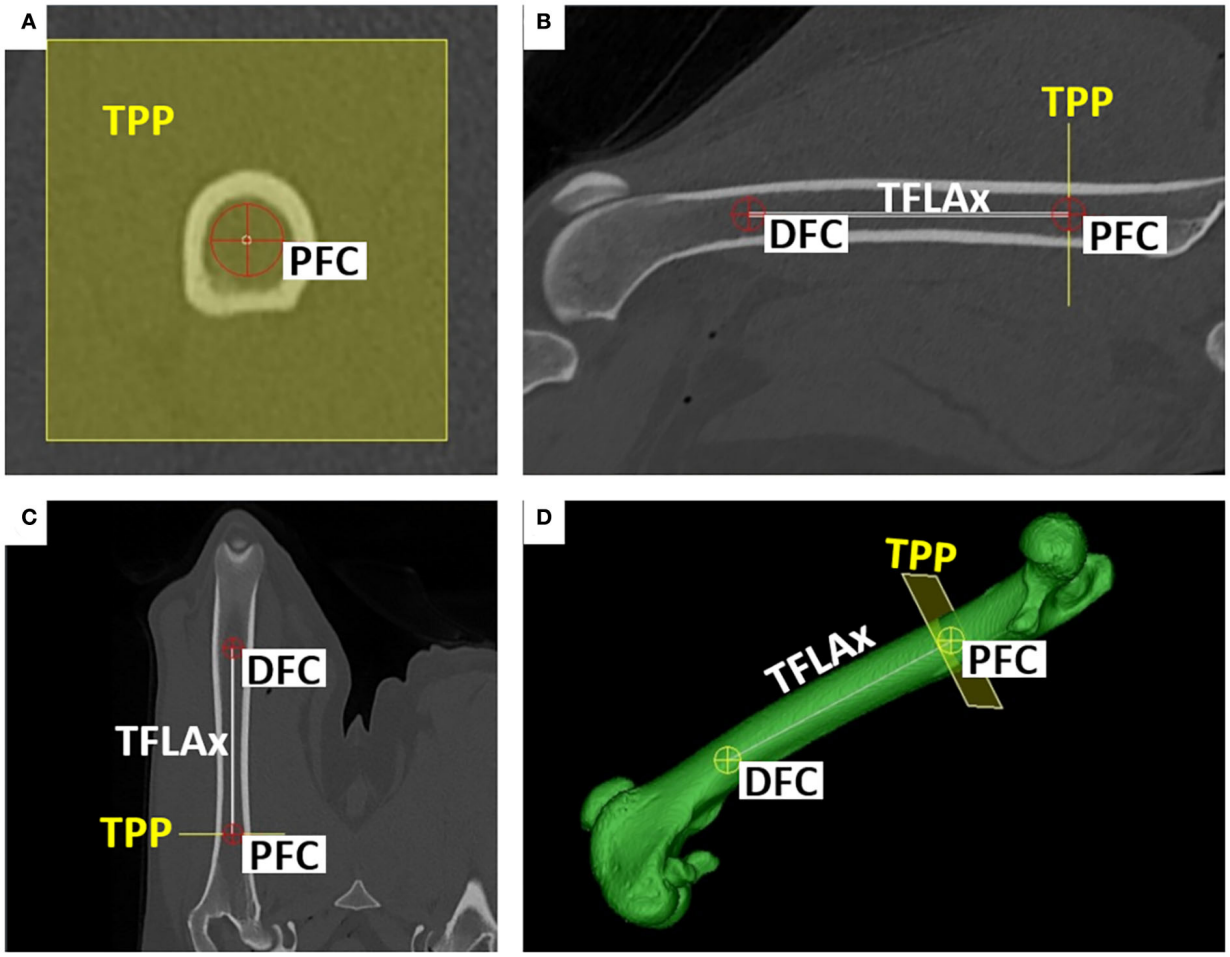
Total femoral longitudinal axis (TFLAx) and transverse projection plane (TPP). Proximal (PFC) and distal femoral shaft centers (DFC) defined the total femoral longitudinal axis (TFLAx). For the calculation of the torsion angles, the transverse projection plane (TPP) was mathematically defined by orthogonality to the total femoral longitudinal axis (TFLAx). Reference points (PFC, DFC) were set and checked in the transverse **(A)**, sagittal **(B)**, dorsal **(C,D)**, and 3D VR images **(D)**.

**Figure 8:**
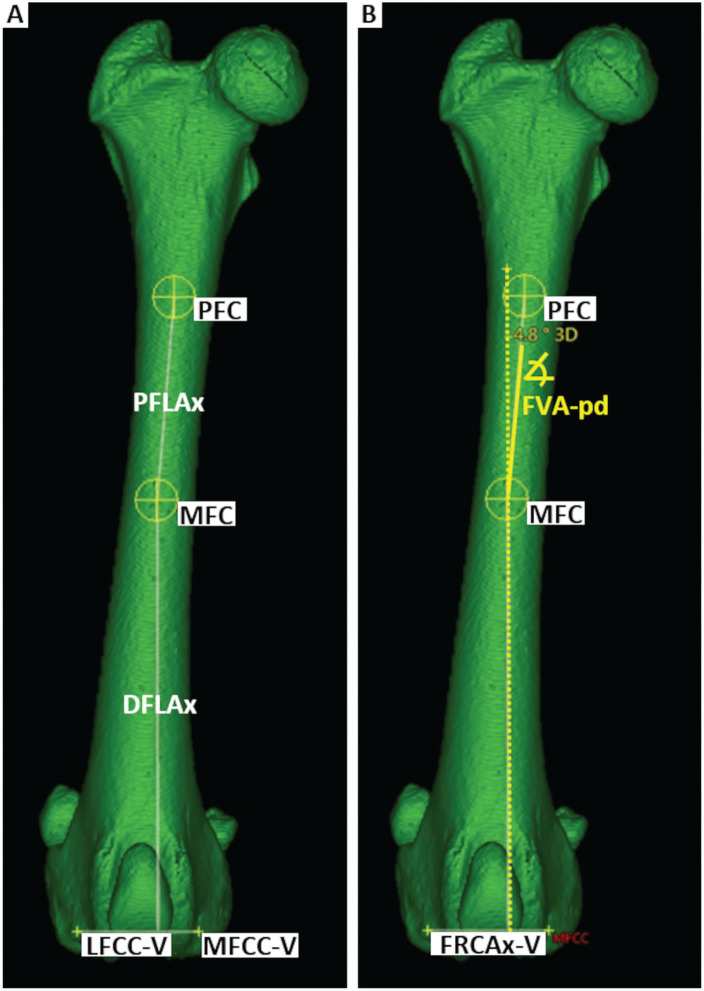
Femoral varus angle (FVA). The proximal (PFC) and mid-diaphyseal (MFC) femoral shaft centers defined the proximal femoral longitudinal axis (PFLAx). The distal femoral longitudinal axis (DFLAx) was defined by orthogonality to FRCAx-V within the dorsal plane **(A)**. The femoral varus (or valgus) angle proximal-distal (FVApd) between PFLAx and DFLAx is calculated within the dorsal plane **(B)**.

Femoral neck axes and femoral retrocondylar axes are oblique and not intersecting skew lines. For the calculation of a femoral torsion angle, both skew lines were projected into a conjoined *transverse projection plane* (TPP). A *total femoral longitudinal axis* was introduced to define the transverse plane via orthogonality (Figure 7). For the definition of a longitudinal femoral axis, additional anatomical reference points were set. The *proximal femoral shaft center* (PFC) was placed in the midpoint of the proximal femoral diaphysis using an encircled crossline tool. This point was located at the transition from the proximal to the middle third of the overall femur length, and at the level where the proximal square-cut appearance of the femoral transverse cross-section becomes round, scrolling distally. The *distal femoral shaft center* (DFC) was placed in the midpoint of the distal femoral diaphysis at the transition from the distal to the middle third of the overall femur length using an encircled crossline tool. More precisely, in transverse plane images scrolling distoproximally, the distal femoral shaft center (DFC) was set at the level where the distal square-cut appearance of the femoral transverse cross-section became round, proximal to the popliteal surface and the supracondylar tuberosities. The *total femoral longitudinal axis* (TFLAx) connected the proximal femoral shaft center and the distal femoral shaft center, and defined the *transverse projection plane* via orthogonality for the calculation of both variants of femoral torsion angles (Figure 7). For the calculation of the varus (or valgus) angle a *proximal femoral longitudinal axis* (PFLAx) was determined by the proximal femoral shaft center (PFC) and a mid-diaphyseal femoral shaft center (MFC) that was placed in the midpoint of the femoral shaft using an encircled crossline tool, dividing the total femur length into two equal parts, using a measuring grid. The *distal femoral longitudinal axis* (DFLAx) was perpendicular to the *femoral retrocondylar axis-varus* in the dorsal plane (Figure 8).

#### Bone-centered 3D coordinate system

**Figure 9:**
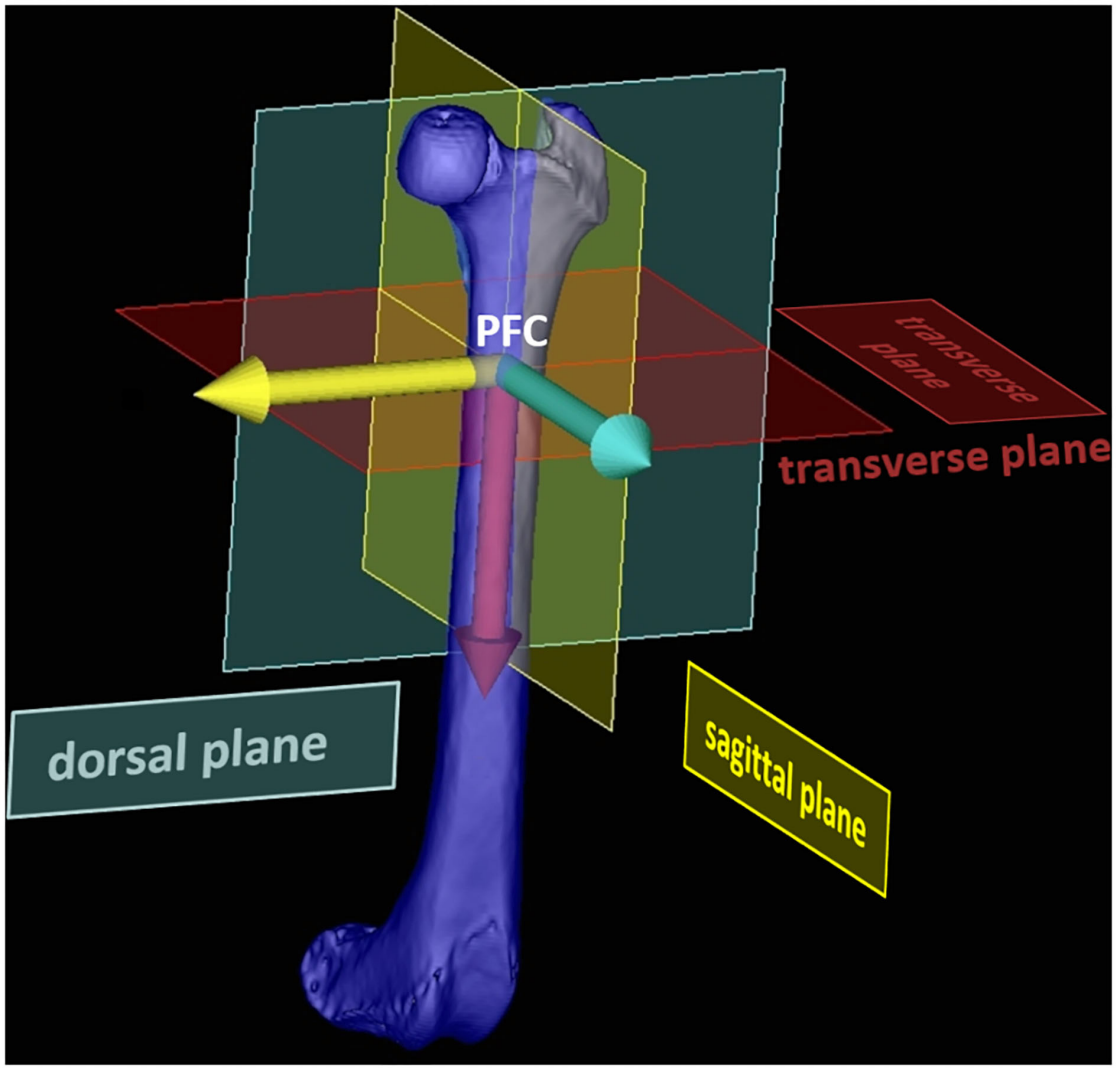
Bone-centered femoral 3D coordinate system (FCS). The femoral 3D coordinate system was centered within the diaphysis of the bone by using the proximal femoral center (PFC) as the origin. The transverse plane was defined by orthogonality the total femoral longitudinal axis (red arrow). Parallel translation of the femoral retrocondylar axis varus the total femoral longitudinal axis, mathematically defined the dorsal plane (yellow and red arrow). The three axes (red, yellow, and blue) originate perpendicular to each other at the point of origin (PFC). This aligned the coordinate system so that its mathematical axes corresponded to the anatomical planes of the femur.

Using the 3D coordinates of each reference point, the femoral torsion angles, the femoral neck inclination angles and the varus (or valgus) angles could be calculated based on vector geometry. A bone-centered 3D Cartesian coordinate system for each bone was introduced. The point of origin of the bone-centered *femoral coordinate system* (FCS) was the proximal femoral shaft center (Figure 8). The proximal and distal femoral shaft centers (total femoral longitudinal axis) defined the first axis of the coordinate system. Parallel translation of the femoral retrocondylar axis-varus to the femoral longitudinal axis defined the dorsal plane of the bone. Orthogonally, transverse and sagittal planes intersected at the level of the proximal femoral shaft center and fully defined a femoral 3D coordinate system having x-, y-, and z-axes (Figure 9).

#### Geometric calculation of the femoral angles

##### Femoral torsion angle (syn. femoral neck version angle)

**Figure 10:**
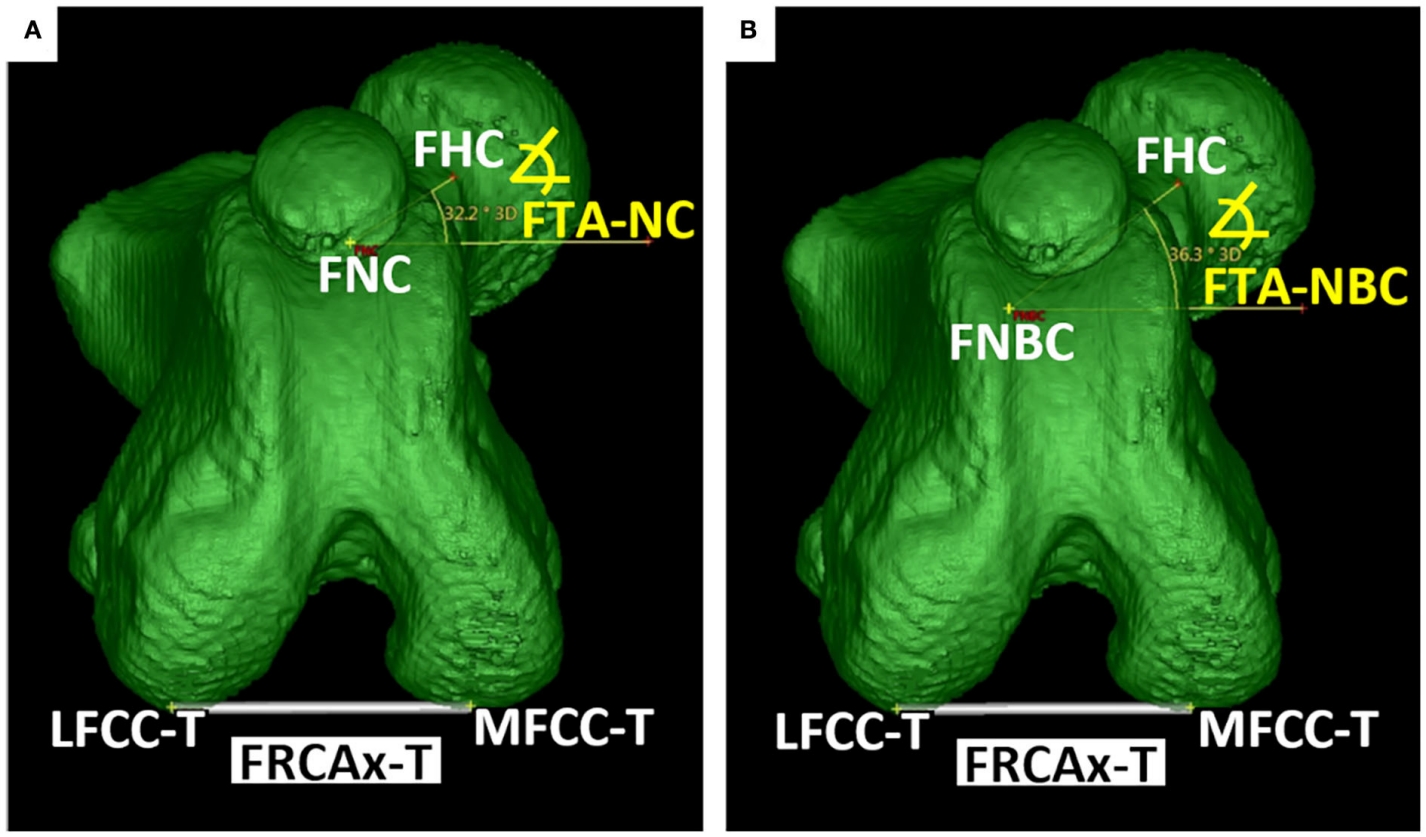
Femoral torsion angles (FTA). For the measurements of the torsion angles, the proximal and distal axes (oblique skew lines) were projected into the transverse plane, to create an intersection and angle. Femoral torsion angle-neck center (FTA-NC) was calculated using the axis between the femoral head (FHC) and neck center (FNC) **(A)**. Femoral torsion angle-neck base center (FTA-NBC) was calculated using the axis between the femoral head (FHC) and neck base center (FNBC) **(B)**. The lateral (LFCC-T) and medial (MFCC-T) femoral condyle centers defined the femoral retrocondylar axis (FRCAx-T) **(A,B)**.

For the calculation of the femoral torsion angles, the femoral neck axes and the femoral retrocondylar axis-torsion needed to intersect. The software dropped perpendiculars from the proximal and distal femoral reference points into the transverse plane and measured the angles between the projected intersecting axes. Femoral torsion angles were calculated in two alternative variations. The femoral neck axis-neck center was projected into the transverse plane for the calculation of the *femoral torsion angle-neck center* (FTA-NC). The femoral neck axis-neck base center was projected into the transverse plane for the calculation of the alternative *femoral torsion angle-neck base center* (FTA-NBC) (Figure 10).

##### Femoral neck inclination angle (cervicodiaphyseal angle)

**Figure 11:**
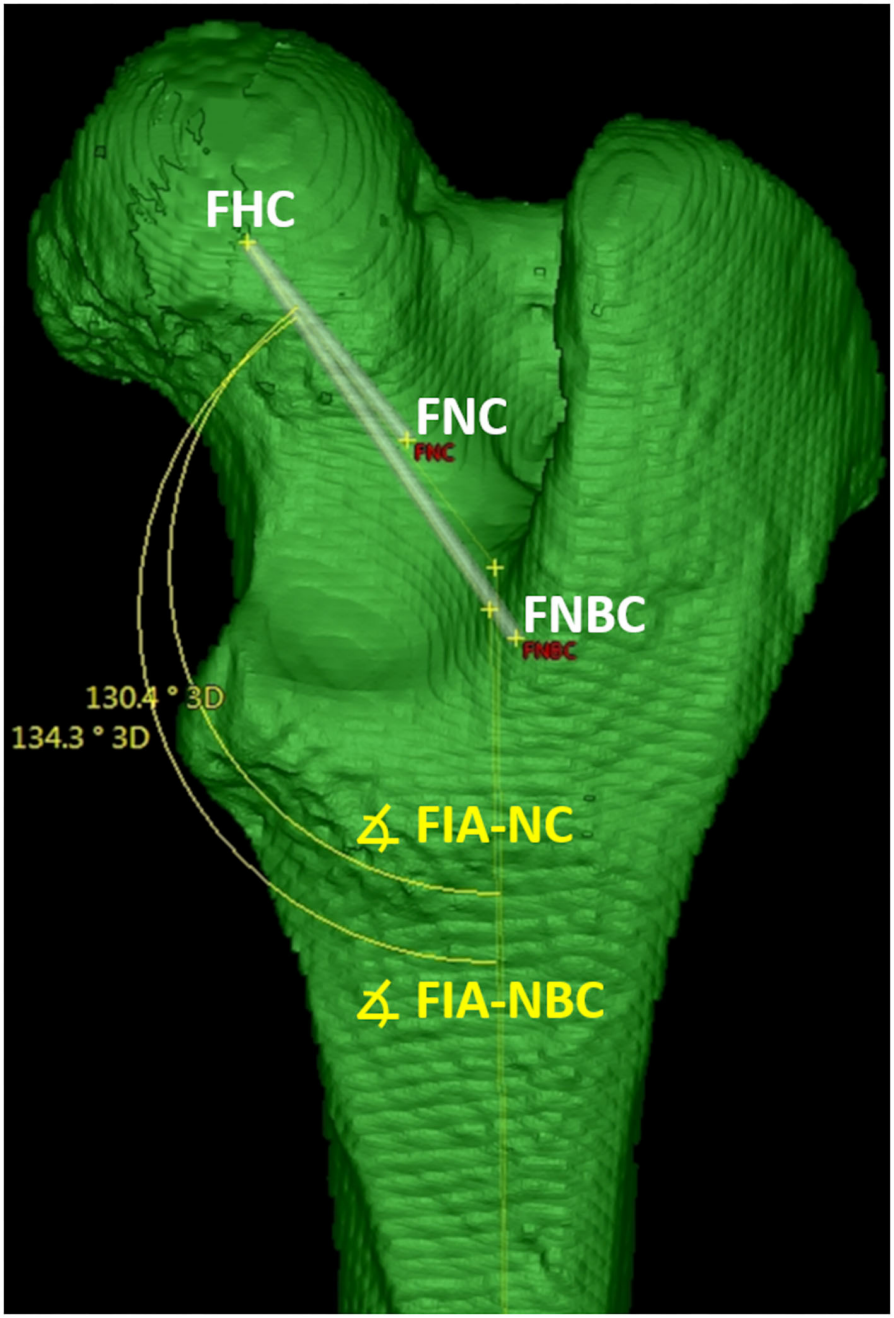
Femoral (Neck) inclination angles (FIA). The femoral neck inclination angle-neck center (FIA-NC) was calculated using the axis between the femoral head (FHC) and neck center (FNC). The femoral neck inclination angle-neck base center (FIA-NBC) was calculated using the axis between the femoral head (FHC) and neck base center (FNBC). The femoral neck inclination angles were calculated in a projection plane defined by the femoral head center (FHC) and the total femoral longitudinal axis, which was also the second axis for angle measurement.

Femoral neck inclination angles were calculated in two alternative variations. *Femoral neck inclination angle – neck center* (FIA-NC) was calculated using the *femoral neck axis-neck center* between the femoral head and neck center. The alternative *femoral neck inclination angle – neck base center* (FIA-NBC) was calculated using the alternative *femoral neck axis-neck base center* between the femoral head and neck base center. The femoral neck inclination angles were calculated in a projection plane mathematically defined by the femoral head center and the total femoral longitudinal axis, which was also the second axis for angle measurement (Figure 11).

##### Varus angle

Parallel translation of the femoral retrocondylar axis-varus into the dorsal plane of the femoral coordinate system, and a perpendicular to the femoral retrocondylar axis-varus, defined the distal femoral longitudinal axis (DFLAx). In the dorsal plane, the femoral varus (or valgus) angle was calculated in two alternative variations. Both were based on the distal femoral longitudinal axis (DFLA), intersecting, first with the total femoral longitudinal axis (TFLAx) for the *femoral varus angle total-distal* (FVA-td) and second with the proximal femoral longitudinal axis (PFLAx) for the *femoral varus angle proximal-distal* (FVA-pd).

An angle of 0° between the total or proximal and distal femoral axes was considered straight and was scaled to 180° to avoid positive and negative values for the statistical calculations. Lateral deviation of the distal femoral axis was considered *valgus* (lateral angle opening), and the resulting value was added to 180° so that angles >180° were valgus angles. Medial deviation of the distal femoral axis was considered *varus* (medial angle opening), and the resulting value was subtracted from 180° so that angles <180° were varus angles. Femoral reference points, axes, angles, and abbreviations are summarized in Table 1.

**Table 1:** Summary of femoral reference points, axes, and angles.

**Reference points**	
Femoral head center	FHC
Femoral neck center	FNC
Femoral neck base center	FNBC
Proximal femoral shaft center	PFC
Mid-diaphyseal femoral shaft center	MFC
Distal femoral shaft center	DFC
Lateral femoral condyle center-caudal for torsion (T)	LFCC-T
Medial femoral condyle center-caudal for torsion (T)	MFCC-T
Lateral femoral condyle center-distal for varus or valgus (V)	LFCC-V
Medial femoral condyle center-distal for varus or valgus (V)	MFCC-V
**Axes**	
Femoral neck axis-neck center / (FHC-FNC)	FNAx-NC
Femoral neck axis-neck base center / (FHC-FNBC)	FNAx-NBC
Femoral retrocondylar axis-torsion (T)	FRCAx-T
Femoral retrocondylar axis-varus (V)	FRCAx-V
Total femoral longitudinal axis	TFLAx
Proximal femoral longitudinal axis	PFLAx
Distal femoral longitudinal axis	DFLAx
**Angles (femoral torsion angle syn. femoral neck version angle)**	
Femoral torsion angle-neck center / (FHC-FNC)	FTA-NC
Femoral torsion angle-neck base center / (FHC-FNBC)	FTA-NBC
Femoral neck inclination angle-neck center / (FHC-FNC)	FIA-NC
Femoral neck inclination angle-neck base center / (FHC-FNB)	FIA-NBC
Femoral varus or valgus angle total-distal (between DFLAx and TFLAx)	FVA-td
Femoral varus or valgus angle proximal-distal (between DFLAx and PFLAx)	FVA-pd
**Coordinate system and planes**	
Femoral coordinate system	FCS
Transverse projection plane	TPP
Dorsal projection plane	DPP

### Evaluation of the 3D technique

A truly 3D technique should always provide the same angle measurement results, no matter how the bone is positioned in the CT. Therefore, independence of positioning was tested by repeated scans of the same bones in different straight and various oblique positions in the gantry. For the experiment, 13 normal canine femur bones were temporarily borrowed from the teaching material collection of the Institute of Veterinary Anatomy.

#### Independence of bone position in the CT gantry

**Figure 12:**
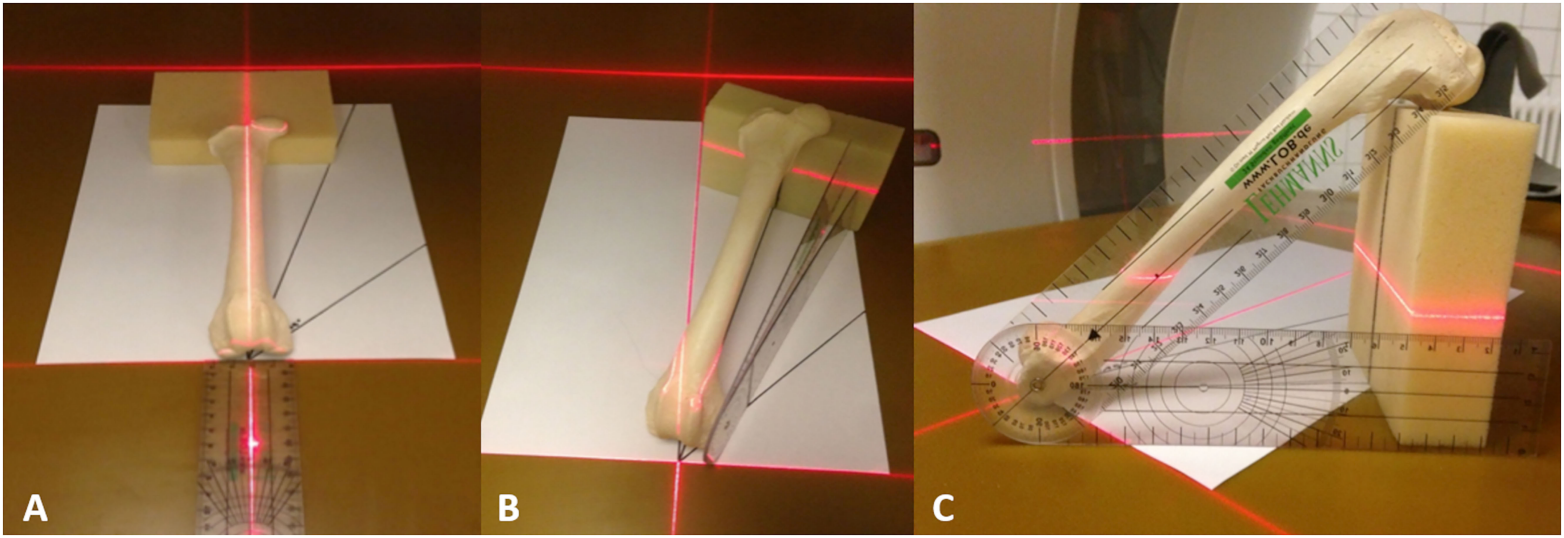
Femur positioning. Parallel (0^**°**^) to the z-axis **(A)**, 15^**°**^ **(B)**, and 45^**°**^ **(C)** double obliquely deviated positioning of the femurs on the CT table in the gantry for scanning to test for the independency of femoral positioning.

Each femur (*n* = 13) was scanned in three different positions. For the first CT scan, all 13 bones were positioned with their longitudinal axis parallel to the z-axis of the CT scanner. For the second CT scan, the bones were positioned double oblique to the z-axis, with 15° deviations in the directions of the x- and y-axes. For the third CT scan, the bones were positioned double oblique to the z-axis, with 45° deviations in the directions of the x- and y-axes. Bones were aligned on the CT scanner table using the three orthogonal positioning lasers of the CT scanner gantry and a goniometer (universal manual transparent plastic full circle 360° with 1° readout increment, Rulongmeter style) (Figure 12). All 39 CT scans were performed by one operator with a helical multi-slice CT scanner with a fixed detector array design (Somatom Definition AS VA48A_02_P12, 64 Excel Ed. software Somaris/7 syngo CT VA48A Siemens Healthcare GmbH, Erlangen, Germany) in a helical mode. Scanning slice thickness was 0.6 mm, tube voltage 120 kV, tube rotation time 1 s, spiral pitch factor 0.8, and X-ray tube current was 96 mA. The reconstructed slice thickness and increment were 0.6 mm. Images were reconstructed using a bone algorithm (deconvolution filter: kernel 70). From the CT scanner, DICOM files of the CT images were exported to a network-attached storage and imported to the medical imaging software VoXim^®^ (version 6.5.1.1 (T2160910) Copyright) with templates prototyped for this study. Femoral varus (or valgus), neck inclination, and version (torsion) angles were measured for each bone and each position independently. When setting the reference points, one was blinded with regard to the resulting angles. The results between different straight and oblique positions of the bones during the scan were compared by subtraction of their means to demonstrate the independence of positioning of the 3D measurement technique.

#### Precision

The precision of the 3D technique was tested by repeated measurements. Two independent persons measured femoral varus (or valgus), neck inclination, and version (torsion) angles in all 39 CT scans. One person (observer 1) measured the angles two times on two occasions, 6 weeks apart. During the second measurement, the results of the first measurement were not visible. A second person (observer 2) performed the measurements, independently from the first observer. The angle measurement results of observer 1 were compared between his two occasions (intra-observer agreement). The results of observer 1 on his first time were compared with the results of observer 2 (inter-observer agreement).

#### Statistical analysis

If the angle measurements are independent of the bone positioning in the scanner gantry, then angle calculations based on the 3D coordinates should always provide the same measurement results. Therefore, we subtracted the mean angle measurement results for each angle and bone position in each possible combination (angle results at 0° minus angle results at 15°, angle results at 0° minus angle results at 45°, and angle results at 15° minus angle results at 45°). When subtracting the mean measurement results for each angle between CT datasets with differently positioned bones, the differences in the subtractions should tend to be zero. For calculating the BIAS and its *p*-value for each angle, the mean differences between the parallel (0°) and each double oblique off-z-axis (15° and 45° off-z-axis deviation) positioning of the femurs on the CT-table in the gantry during scanning were calculated according to Bland and Altman [Bland JM, Altman DG. Statistical methods for assessing agreement between two methods of clinical measurement. Lancet. 1986 Feb 8;1(8476):307-10. PMID: 2868172] (72). To estimate the repeatability (intra- and inter-observer variability) of femoral angular measurements, the *Coefficient of Variation (CV) for repeated measurements* was calculated according to Bland 2000 [Bland M. An introduction to medical statistics. Oxford University Press 3. Ed. P 269-272] (73). To estimate the intra-observer variability, the *CV for repeated measurements* of the same observer was calculated. To estimate the inter-observer variability, the *CV for repeated measurements* was calculated for the same measurements of different observers. *CVs for repeated measurements* were considered excellent <3%, good <10%, moderate/fair <15%, and poor >15%. The statistical analysis was performed by using the software IBM SPSS 23 and MedCalc 20.111.

## Results

For all femoral CT scans, the operator was able to visually evaluate the scans and place reference points into the images based on anatomical localization using MPR and VR. Overlays of selected reference points, axes, angles, and planes allowed for monitoring of the process. The program could calculate all angles. Therefore, we considered this technique feasible. Application of the method and the use of the templates required initial training of the operator.

The test for independence from the positioning of a normal canine femur on the table by double oblique deviation from the z-axis (Figure 12) in the scanner gantry was expressed by the mean differences between the measurements of various double oblique off-angle scans of the femur and resulted in mean differences between −0.9° and 1.3° for all six determined types of angles and also resulted in a difference of <1° for 17 out of 18 calculations (with one outlier of 1.29°, *p*-value 0.002, femoral neck inclination angle-neck base center) as shown in Table 2.

**Table 2:** Mean differences between angular measurement results of parallel (0**°**) and varying off-z-axis (15**°** and 45**°** deviated) double oblique positioning of the femurs on the CT table in the gantry during scanning to test for the independency of femoral positioning.

**Femurs: *n* = 13** **Positioning: Three times** **CT-scans: 39**	**Subtraction of the results of different positioning angles**	**Mean**	***p*** **-value**
Femoral torsion angle-neck center	FTA-NC (0°) - FTA-NC (15°)	−0.626	0.075
(FTA-NC)	FTA-NC (0°) - FTA-NC (45°)	−0.933	0.201
	FTA-NC (15°)–FTA-NC (45°)	−0.308	0.362
Femoral torsion angle-neck base	FTA-NBC (0°)–FTA-NBC (15°)	−0.400	0.145
center (FTA-NBC)	FTA-NBC (0°)–FTA-NBC (45°)	−0.523	0.096
	FTA-NBC (15°)–FTA-NBC (45°)	−0.123	0.712
Femoral neck inclination angle-neck	FIA-NC (0°)–FIA-NC (15°)	−0.662	0.124
center (FIA-NC)	FIA-NC (0°)–FIA-NC (45°)	−0.813	0.170
	FIA-NC (15°)–FIA-NC (45°)	−0.151	0.644
Femoral neck inclination angle-neck	FIA-NBC (0°)–FIA-NBC (15°)	0.659	0.131
base center (FIA-NBC)	FIA-NBC (0°)–FIA-NBC (45°)	1.29	0.002
	FIA-NBC (15°)–FIA-NBC (45°)	0.628	0.163
Femoral varus or valgus angle	FVA-td (0°)–FVA-td (15°)	0.359	0.089
total-distal (FVA-td)	FVA-td (0°)–FVA-td (45°)	0.364	0.055
	FVA-td (15°)–FVA-td (45°)	0.005	0.966
Femoral varus or valgus angle	FVA-pd (0°)–FVA-pd (15°)	0.449	0.015
proximal-distal (FVA-pd)	FVA-pd (0°)–FVA-pd (45°)	0.31	0.154
	FVA-pd (15°)-FVA-pd (45°)	0.139	0.283

Intra-observer agreement determined by repeated femoral measurements by the same person on two different occasions resulted in coefficients of variation for repeated measurements below 4% and parallel independent measurements by two different observers resulted in coefficients of variation for repeated measurements below 5.5%, except for the femoral torsion angle-neck center, that had a higher coefficient of variation for repeated measurements (13.5%), as shown in Table 3. Measurements of the two torsional angles resulted in coefficients of variation for repeated measurements between 1.5 and 13.5% which were higher than those for femoral neck inclination and varus or valgus angles that ranged between 0.2 and 3.2%. Determination of intra-observer agreement resulted in coefficients of variation for repeated measurements between 0.2 and 3.9% which were lower than those for the inter-observer agreement which ranged between 1.3 and 13.5%, as shown in Table 3. All individual results of the 702 angular measurements in 39 CT scans of 13 normal canine femur bones that were scanned in different positions on the scanner table to test for the independency of femoral positioning and reproducibility of femoral angular measurements are listed in Supplementary Table 1.

**Table 3:** Reproducibility of femoral angular measurement results of various parallel (0°) and varying off-z-axis (15° and 45° deviated) double oblique positioning of the femurs on the CT table in the gantry during scanning to test for the independency of femoral positioning.

**Femurs: n = 13** **Positioning: Three times** **CT-scans: 39**	**Intra-observer agreement**			**Inter-observer agreement**		
**Angle**	**Mean**	**Standard deviation**	**Coefficient of variation for repeated measurements (%)**	**Mean**	**Standard deviation**	**Coefficient of variation for repeated measurements (%)**
Femoral torsion angle-neck center (FTA-NC)	17.913	0.699	3.899	18.509	2.49	13.454
Femoral torsion angle-neck base center (FTA-NBC)	26.931	0.394	1.464	26.537	1.459	5.499
Femoral neck inclination angle-neck center (FIA-NC)	124.769	0.4891	0.392	126.26	4.011	3.177
Femoral neck inclination angle-neck base center (FIA-NBC)	140.912	0.936	0.664	141.055	2.012	1.426
Femoral varus or valgus angle total-distal (FVA-td)	180.142	0.376	0.209	179.103	2.628	1.467
Femoral varus or valgus angle proximal-distal (FVA-pd)	179.264	0.396	0.221	178.987	2.338	1.306

## Discussion

### Three-dimensionality

#### 3D VR CT

VR CT allows free rotation of bones resembling virtual radiographic positioning, which is considered three-dimensionality (13, 14, 17, 21, 22, 25, 43, 46, 47, 55–57, 59–63). The operator visually selects a 3D VR bone position and view to finally measure an angle within a flat image that lacks the third dimension. Oblique skew lines in the 3D bone anatomy become coplanar in the 2D image and the creation of this projection enables angular measurements. There are numerous possible 2D image perspectives on a 3D bone. The operator's visual selection of a view intended for measurement can be standardized in a normal bone. However, with a bone deformed in different planes and directions, the visual selection of bone position and perspective is difficult to standardize and remains subjective. Therefore, and despite the use of 3D VR CT, angle measurements are problematic and variable in severe and complex angular and torsional deformities (13).

#### Mathematical standardization of virtual bone positioning

In this study, we have replaced the visually guided virtual 3D positioning of VR CT bone images with a mathematical definition. We introduced a total femoral longitudinal axis and centered the 3D coordinate system into the diaphysis of the bone. We aligned the orientation (x, y, z) of the bone-centered 3D coordinate system in 3D space with the three anatomical planes (sagittal, transverse, and dorsal) of the bone. This allowed the projection of axes and angles directly over the bones and provided an overview for the operator. Axes and projection planes could have been defined differently. Mathematics and vector calculus open up many new possibilities here. To be able to set reference points not only in a single post-processed VR CT image but also within multiple images and at arbitrary positions in the whole data set, the 3D coordinates of the reference points were used. Anatomical reference points that were used in human medicine (24) and for the canine femur (38, 39, 74) were described with the parallel use of MPR and VR. Instead of using only one CT image post-processing method, MPR or VR, we consider the parallel and complementary use of both methods to be advantageous for precise reference point placement. The use of 3D coordinates of reference points in 3D space and 3D mathematical definition of projection planes for angular measurements made the method truly three-dimensional and therefore totally independent of femoral positioning. A few studies on the canine hind limb report use similar approaches: CAD software enabled true 3D measurements in 3D models of optical scans of normal canine femoral cadaver bones (30). Using DICOM software, proximal and distal fiducial markers were introduced into the diaphysis to standardize the virtual positioning of canine 3D VR CT images for torsion angle measurements in normal canine tibiae (75).

### Potential advantages and evaluation benchmarks

In a straight normal bone, this 3D method might not bring an advantage compared to 3D VR CT. It may not even give a benefit over a perfectly positioned radiograph. Presumably, the 3D technique provides better accuracy in severely deformed bones. But this 3D technique, as well as other methods described for normal bones (15–32) must finally prove their accuracy in deformed bones, which is difficult. We have not yet compared CT and goniometer measurements on bone specimens to assess true anatomic accuracy. In the laboratory, normal bone specimens were used to compare radiological measurements with anatomical measurements (15, 22, 28, 37, 75). Nevertheless, it is difficult to establish a gold standard, because an anatomical measurement of a bone with a goniometer in a laboratory is not necessarily more precise or accurate than a radiological measurement. Ideally, a measurement is perfectly accurate and precise. The precision of a measurement only does not prove its accuracy. The requirements for a technique in terms of accuracy and precision are not specifically defined, to the best of our knowledge. To be able to propose a benchmark and apply an evaluation standard in terms of precision for a measurement technique, it could be compared to the precision of an orthopedic procedure, such as a corrective osteotomy. Quantitative assessment of the inaccuracy of angular manipulation in a human cadaver study revealed a mean error of 8.8° for Kirschner wire placement using a manual goniometer technique, which could be reduced to 2.1° mean error when a mounted digital goniometer device was used (76). In our study, to determine precision (intra- and inter-observer agreement), we performed repeated measurements. To determine accuracy in terms of three-dimensionality and independency of positioning, we performed repeated scanning in different bone positions up to 45° oblique inclination. Additional errors and variations due to manual goniometer measurements used for the oblique positioning of the bones in our experiment can be assumed, probably in a similar range as in other studies using goniometer measurements (76). Comparing the measurements of parallel and oblique scans by subtracting their results showed differences that tended to zero and did not exceed 1.3°. Considering an oblique inclined position up to 45°, if the measurements were not 3D, erroneous, or biased, greater differences than 1.3° should be expected. Furthermore, 1.3° is less than the current manually achievable surgical accuracy (76).

### Restriction of reference values

Limitations of 2D radiography have been pointed out, its measurement results have been questioned and CT was recommended for determining canine femoral torsion angles (57). Multiple individual studies on femoral angular measurements of dogs using different imaging modalities and measurement techniques have been compiled and compared (57, 58). The need for reference values for the canine femoral alignment has been noted (58). In human medicine, CT reference values for femoral alignment angles are not universally applicable but are only specifically valid for the respective CT measurement technique used (77, 78). This is probably also true for canine femoral alignment in veterinary medicine. Apart from the use of different measurement techniques, reference values could vary with dog conformation or might even be breed-specific (58). Assuming specificity in terms of dog breed and measurement technique, any reference value for femoral torsion should be used with caution. Values from this study should therefore not be used as clinical guidance. Reference values are unlikely to be universally applicable when different imaging modalities and measurement techniques are used, and they differ between 2D and 3D methods (30).

### Limitations of this 3D method at the current stage and further steps

#### Only femur—Not tibia nor whole limbs

We have described a 3D measurement procedure for the canine femur only. Hindlimb deformities affect not just the femur but also the tibia. Therefore, a measurement method should not be limited to the femur, but must look at the dog's hindlimb as a whole, that is, all bones and joints and their relationship and contribution to the overall alignment of the hindlimb. The 3D measurement techniques for all bones and joints would be desirable in future. CT measurement techniques of the dog are based on those in human medicine (24, 47, 56, 79). In men, the position of the legs, especially the extension in the hip and knee joints, is relatively similar when standing and lying at the CT table. The human knees are extended to 180°, femur and tibia lie on one axis. This is different in the quadruped canine patient. In dogs, the coxofemoral, stifle, and tarsal joints cannot be extended to 180°, and are therefore never aligned along a straight axis during the scan. Inaccuracy during positioning may increase in patients with limited joint mobility due to osteoarthritis or muscle contraction, and in patients with severe bone deformities or fracture deformities, in whom the accurate determination of three-dimensional limb alignment is of particular interest (46). The varying degree of caudal extension of the hind limbs leads to variations in positioning and oblique transverse cross sections, which are a potential source of erroneous angle measurements in 2D techniques. Based on the goal to fully examine both hind legs in one CT scan and the limitation that the tibia and femur cannot be aligned in one straight axis, the goal was to make the technique independent of femoral positioning. Due to positional independence, a 3D measurement technique of the tibia can be easily added.

#### Torsion and varus angles only—No sagittal plane deformities

In this project, we described 3D measurements of torsion angles in the transverse plane and varus or valgus angles in the dorsal plane of the femur. Bone deformities are not limited to these two types of deviation, but also occur in the sagittal plane. A comprehensive method should include measurements in all three planes. In future, this 3D method must be extended to include the deformities in the sagittal plane that occur as procurvatum and recurvatum (46).

#### Isolated normal femurs only—No patients with deformed bones

We have described a 3D method and have demonstrated that it can measure angles independent of position in normally isolated femurs. Many techniques have initially been described on normal cadavers and normal bones (15–32). However, femoral angle measurements are especially interesting in canine patients with patellar luxation (1, 3, 5–7, 19, 33–45) and severe posttraumatic bone deformity (4, 13, 46, 47). A technique that has proven successful in normal femurs has not yet proven its feasibility in deformed bones. Further testing is required to evaluate the precision and accuracy of this method in normal and deformed patient bones. Currently, the use of this technique has not been described for deformed bones in canine patients. Therefore, this technique cannot yet be recommended for clinical use until its successful application in clinical patients with bone deformities has been demonstrated. Type, severity, and combinations of osseous deformities might influence whether a technique could be successfully applied or not. Difficulties might occur because of changes or deformation of the anatomical measurement points caused by posttraumatic bone callus formation, malunion, translational dislocation, periosteal reaction, exostosis, periarticular osteophytosis, osteoarthritis, dysplasia, or other anomalies involving limb malformations. Further evaluation requires application in clinical cases with severe and complex three-dimensionally deformed bones, for which we have developed this 3D method.

### Software platform

The need for image data export from the CT scanner or image archive and import into an additional program is a disadvantage of this technique. VoXim^®^ was a commercial medical device-approved 3D imaging software (68–71) that was based on the DICOM (Digital Imaging and Communications in Medicine) technical standard (80, 81). Commercial software is a disadvantage of this study due to its cost, until a free open-source variant of this technique is available. Medical imaging software for use on humans usually requires approval as a medical device by national authorities. Currently in most countries, to the authors' knowledge, neither regulatory approval for medical devices nor legal licensing is required for the use of software for diagnostic imaging in animals. Veterinarians can use viewer software without medical device approval and even other technical imaging standards and platforms, such as computer-aided design (CAD) software (25, 30, 82). We consider the use of a DICOM-based viewer to be advantageous due to its interoperability. DICOM is the technical standard commonly used in veterinary diagnostic imaging (80, 81). Software engineers and companies in the field of veterinary medicine might be inspired to develop imaging tools for true 3D measurements. As an alternative to commercial programs, open-source software for a 3D measurement method would be desirable. This would enable researchers and software programmers in collaboration with practicing orthopedic surgeons to further develop the method in a decentralized and collaborative way.

### Software tools and automatization

This 3D method adopted a semi-automatic approach. To improve the accuracy of the determination of the reference points, eyeballing was supported by several tools. The femoral head fitting sphere was automatically triggered by a polyline consisting of manually set reference points. A superimposed measurement grid helped to divide and compare distances. An encircled crossline tool assisted in precisely spotting the center of a round or squared cross-section. An improvement for this feature would be a variable setting option for size, line thickness, or dotted lines. Reference points were set at bone surfaces and within bones. VR views provide a good overview which is helpful for reference points at the bone surface. Since VR images only display the surface, they are not suitable for setting intraosseous points. Cross-sectional images are well-suited for placing points within the bone. We combined both, VR and three-plane orthogonal MPR, to maximize accuracy. Free 3D MPR and curved MPR tools would be further improvements. Overlays created by the projection of intraosseous reference points and axes onto the surface of semitransparent free VR images enhance overview and enable plausibility checks. In MPR mode, reference points located directly within the current image could be distinguished from reference points overlaid by adjacent cross-sections, by means of a color code.

User-friendliness and measurement times were not evaluated in this study, which is a drawback. However, the templates were not final products for the end user at this stage. Integration of an illustrated guide into the software could improve intuitive and user-friendliness. Improvement of existing and introduction of new tools, reference points, axes, and angles could extend and improve the semi-automatic 3D measurement method in future. Fully automatic detection of all reference points to automatically measure all angles by recognition of bone shapes and surfaces has already been described for normal isolated bones with CAD software (30). In future, this automated technique probably will replace manual measurements, once it is proven that it can be used successfully in canine patients with bone deformities.

### Anatomical reference points

Detailed anatomic description of the reference points, axes, and projection planes, as well as geometric determination of the angles within the coordinate system, were goals of the study to precisely define the method and make the measurements consistently reproducible. The anatomical location of the reference point, its selection procedure, the technical tools used, and the mathematical definitions of the projection planes for the angular measurements are likely to influence the final angular values. Therefore, these technical aspects require detailed discussion.

#### Femoral head and neck

In the original two-dimensional radiographic technique, the femoral neck axis was defined by a line bisecting the femoral neck and head (18) that was later improved in a CT MPR technique using a circle of best fit (21). Instead of the center of a circle, we determined the 3D coordinates of the *femoral head center* as the midpoint of a 3D ball. A similar 3D approach was used in another truly 3D method based on computer-aided design (CAD) software (30). Their CAD software used fully automated fitting spheres to determine the reference points for the femoral head center and femoral condyle surfaces in three-dimensional data from optical scans of disarticulated and surgically isolated canine cadaver bones (30). VoXim^®^ used a semi-automated fitting sphere. On the surface of the femoral head surface, the operator manually set reference points that were connected by the software to a polyline that triggered the ball. This makes our approach probably more time-consuming, but it should still be possible to set the reference points if the femoral head shape is abnormal due to hip dysplasia and periarticular osteophytes.

For the 3D definition of the femoral neck axis, and to connect with the femoral head center, we required an individual second reference point. We evaluated two alternative reference points with two different femoral neck axes and different technical approaches: *femoral neck axis-neck center* and *femoral neck axis-neck base center*. Originally, in distoproximal radiographs, the center of the femoral neck at its isthmus was used (18). This is similar to our *femoral neck axis-neck center*. The original radiographic technique (18) to determine the femoral head and neck axis was applied in various CT studies (21, 22, 37, 41, 42, 44, 45, 55, 60, 62). A bisecting axis was fitted directly between the centers of the femoral head and neck isthmus, based on a single two-dimensional image using MPR, MIP, or VR (21, 22, 37, 41, 42, 44, 45, 55, 60, 62). Our *femoral neck axis-neck base center* is equivalent to CT studies, in which the center of a transverse slice at the base of the femoral neck, at the level of the lesser trochanter, was used earlier (24, 39). Due to the obliquity and shape of the femoral neck, the *femoral neck center* is difficult to determine in transverse CT images, without the use of free MPR, MIP, and VR. This may be the reason for the use of the *femoral neck base center* in early CT studies (24, 39). The line *femoral neck axis-neck base center* is longer than the line *femoral neck axis-neck center,* where the femoral neck center is at a very close distance to the femoral head center. When two reference points are close to each other, a small spatial deviation in the selection of one point results in a larger influence and a larger change in the value of a geometrically derived angle than when two reference points are farther away from each other. This distance effect could have an impact on the precision. The technical tools used to set the reference points are also likely to have an influence. The *femoral neck base center* was set directly as an individual point and the *femoral neck center* was set indirectly by moving a virtual femoral neck resection plane. From the femoral head center, the course of the perpendicular to the plane at the center of the femoral neck at its isthmus was similar to the axis used in several previous CT studies (21, 22, 37, 41, 42, 44, 45, 55, 60, 62). Our software contained a fixed orthogonal MPR tool that made the direct visualization or setting of this reference point difficult. A freely adjustable double oblique MPR tool or even a curved MPR tool might help and improve determining the femoral neck center in future more easily and consistently. The center of a spherical section in a curved shape along the base of the femoral neck in a transverse orientation to the femoral neck was determined automatically as a “section centroid” in another most recent study (30) as another alternative.

The higher precision of the *femoral torsion angle-neck base center* measurements compared to the *femoral torsion angle-neck center* could be due to the better definability of the femoral neck center, its greater distance from the femoral head center, the difference between the technical tools used, or a combination of these causes. This difference in precision was not so obvious between the measurements of the *femoral neck inclination angle-neck center* and *femoral neck inclination angle-neck base center*. However, the *femoral neck inclination angle-neck base center* was calculated for comparison of precision, and not because it was a real anatomical or clinically relevant angle. The *femoral neck inclination angle-neck center* in this study resembles the true anatomical angle of inclination (23, 50–52) and cervicodiaphyseal angle (28). On a craniocaudal radiograph of a canine femur, the projected angle of inclination is not identical to the true anatomical angle of inclination (23, 50–52) and depends on the view, projection plane, and the angle of version (23, 27, 51). The femoral neck must be parallel to the detector to project the true cervicodiaphyseal angle on a radiograph (28). Even in 3D CT VR images, femoral neck angles depend on and vary with the selected view (25). We standardized this view mathematically. The femoral head center and the total femoral longitudinal axis defined the plane into which the femoral neck axes were projected.

Femoral neck inclination angle measurements showed higher precision than torsion angles. The femoral neck anatomy may allow more precise alignment of the femoral neck axis in the cranial and caudal 3D VR views than in the proximal or distal views, or the user may have paid more attention to them. Other causes might be a higher precision or the higher length of the *total femoral longitudinal axis* compared to the *femoral retrocondylar axis-torsion*. Overlays of intraosseous points and axes on VR images with a free view of perspective were especially helpful for plausibility checks of the femoral neck axes and were considered a useful tool.

#### Femoral condyles

The femoral retrocondylar axes were determined by the lateral and medial femoral condyle centers. In agreement with earlier techniques, these tangents were set caudally for torsion angles (18, 24) and distally for varus angles (15–17). Originally, these points probably came from human medicine (24) and the tradition of available radiographic projections (15–18). In 2D radiographs and VR CT images, these points resemble the condylar subchondral bone surfaces that are hit tangentially by the X-ray beam or perspective image projection at the respective condylar level. The level of the reference point at the femoral condyle results indirectly from the standardization of radiographic positioning (15–17) or in 3D VR CT, a standardized angle of perspective (17). A fitting sphere was used to consistently localize these reference points in normally shaped bones in combination with a standardized joint angle of 90° between the tibial plateau and femoral longitudinal axis (30). Using the femorotibial contact points of a dog in a standing posture (knee joint flexion angle ~ 135°) is another possibility (83). To keep the variation of the measurement results low, we have tried to place the tangent exactly at the same height of the medial and lateral condyle. If the size, shape, and curvature of the medial and lateral condyle are similar, slight proximodistal translation of the tangent should cause less variation of the projected angle than slight obliquity of the tangent. Determining the retrocondylar tangent with the visual sense of proportion at the condylar surface is difficult because there are no landmarks along the condylar curvature. In analogy to the femoral head, intraosseous reference points could also be considered. The pivot axis of the stifle or a tanscondylar axis between the centers of the medial and lateral condyle would be possibilities (84). In 2D images, two different views and therefore two different condylar tangents are used to determine varus (15–17) and torsion (18). With a 3D method, it would be possible to use the same one axis for torsion and varus angle measurements, either a retrocondylar or a transcondylar axis.

### Projection planes

Reference points and reference axes are skew lines that need to be projected into a commonly shared plane. In 2D radiographs or 2D CT images created by MPR or VR techniques, the view and the image itself correspond to the projection plane. Therefore, these techniques heavily rely on the standardization of positioning, beam angulation, or VR view selection. We standardized the projection plane using a geometric definition based on anatomical reference points. Our goal was to make the 3D measurements robust for clinical application in orthopedic patients with hind limb malalignment and bone deformation (2, 3, 6, 44, 45, 85, 86). The projection planes determined by axes were designed in such a way that, hopefully, the method will not fail in difficult orthopedic cases. Surgical candidates for corrective osteotomies are dogs with severe congenital, developmental, or posttraumatic deformities, such as patellar luxation, growth plate injuries, and fracture malunions with bone callus (2, 3, 6, 44, 45, 85, 86). Despite osseous malformation, it should be possible to set the reference points, at least approximately in a suitable place. The measured angles must correlate with the type and degree of deformation. The definition and orientation of the projection plane affect the measurement results.

#### Transverse plane

To define the projection plane for torsion angle measurements, we created a total femoral longitudinal axis defined by a proximal and distal femoral shaft center. As the distance between the reference points increases, the deviation of the course of the line decreases. Therefore, the points should be far apart and the line should be long. Small deviations when setting the reference points do not make a big change in the course of the axis, in the projection of the plane, and thus in the angle measurement. Alternative use of mechanical or anatomical axes would probably be possible as well. Further research is needed to determine which reference points and axes are most appropriate in orthopedic patients with severe bone deformities. The use of an automatic fitting axis along multiple transverse diaphyseal section midpoints is very precise, described for normal femurs (30).

#### Dorsal plane

To mathematically define the dorsal projection plane for varus measurements, we used the orientation of the *femoral retrocondylar axis-varus* in combination with the *total femoral longitudinal axis*. The *distal femoral longitudinal axis* was an orthogonal line to the *femoral retrocondylar axis-varus* within the dorsal plane. This resembles a distal femoral joint orientation similar to many other studies (58). Since we did not have a good solution for a proximal 3D joint orientation line for the femoral head, we decided to create a *proximal femoral longitudinal axis* to measure varus angles in combination with a *distal femoral longitudinal axis*. The use of a *total femoral longitudinal axis* in combination with a *distal femoral longitudinal axis* would probably underestimate a varus deformity whose center of rotation of angulation (CORA) is located between the proximal and distal femoral shaft center. We have calculated two *femoral varus angle* variants (*total-distal* and *proximal-distal*). As expected, the results in this study do not differ very much for straight normal bones. However, we believe that in bones with mid-diaphyseal varus deformities the *femoral varus angle proximal-distal* will provide more representative and accurate results.

## Conclusion

The introduction of a bone-centered 3D coordinate system and the mathematical definition of projection planes based on 3D coordinates of anatomical reference points enabled truly 3D CT measurements of canine femoral torsion and varus angles. The agreement between angle measurement results of bones scanned three times, once in straight and twice in double oblique positions, demonstrated that the technique is independent of femur positioning in the gantry, proving its three-dimensionality.

## Data availability statement

The raw data supporting the conclusions of this article will be made available by the authors, without undue reservation.

## Ethics statement

Ethical review and approval was not required for the animal study because the CT-scan of the dog that was used to prototype the program was retrospectively selected and retrieved from the hospital image archive. Initially, the scan was performed on a dog patient with consent of the owner and the dog had a routine clinical CT scan with a medical indication unrelated to this project. The femoral bones that were scanned to evaluate the software were temporarily borrowed from the veterinary anatomical institute of the LMU Munich and the bones are specimen used for teaching of veterinary students and came from cadavers of dead dogs, that died or were humanely euthanized due to incurable disorders, unrelated to that research project and the canine cadavers were donated by their owners for purposes of teaching and science earlier.

## Author contributions

Conception and study design, development of methodology, measurements and data acquisition (operator 2, observer 2), and analysis and interpretation, as well as draft, revisions, approval, and submission of the article was the work of AB. Measurements and data acquisition (operator 1, observer 1), analysis and interpretation of data, and as well as final approval of the article was the work of BS. CT scans as well as final approval of the completed article were the contributions of MZ. SR selected and calculated the statistical tests, revised the article for intellectual content, and approved the final article. Supervision, revision of the article for intellectual content, and approval of the final article was the work of AM-L. All authors contributed to the article and approved the submitted version.

## Conflict of interest

The authors declare that the research was conducted in the absence of any commercial or financial relationships that could be construed as a potential conflict of interest.

## Publisher's note

All claims expressed in this article are solely those of the authors and do not necessarily represent those of their affiliated organizations, or those of the publisher, the editors and the reviewers. Any product that may be evaluated in this article, or claim that may be made by its manufacturer, is not guaranteed or endorsed by the publisher.

## Supplementary material

Supplementary Table 1 Individual measurement results of the test for independency from femoral positioning.
